# Psychosocial health and psychological adjustment in adolescents and young adults with congenital melanocytic nevi: Analysis of self-reports

**DOI:** 10.3389/fpsyg.2022.911830

**Published:** 2022-08-25

**Authors:** Ornella Masnari, Kathrin Neuhaus, Clemens Schiestl, Markus A. Landolt

**Affiliations:** ^1^Department of Psychosomatics and Psychiatry, University Children’s Hospital Zurich, Zurich, Switzerland; ^2^Children’s Research Center, University Children’s Hospital Zurich, Zurich, Switzerland; ^3^Division of Plastic and Reconstructive Surgery, Department of Surgery, University Children’s Hospital Zurich, Zurich, Switzerland; ^4^Division of Child and Adolescent Health Psychology, Department of Psychology, University of Zurich, Zurich, Switzerland

**Keywords:** psychological adjustment, health-related quality of life, psychosocial health, skin lesion, adolescence, self-reports, congenital melanocytic nevi, birthmark

## Abstract

This study assessed self-reported health-related quality of life and psychological adjustment in 43 adolescents and young adults (ages in years: 14–24, *M* = 17.6, *SD* = 2.2) with congenital melanocytic nevi (CMN) and examined associations with sociodemographic variables, characteristics of the CMN, perceived social reactions, and cognitive emotion regulation strategies. Outcome measures included the Pediatric Quality of Life Inventory^™^ 4.0 and the Strengths and Difficulties Questionnaire. Findings suggest impaired psychosocial health and psychological adjustment in youth with CMN compared to community norms. Impairments were associated with higher age of participants, lower socioeconomic status, visibility of the skin lesion, perceived stigmatization, poorer perceived social support, and maladaptive cognitive emotion regulation strategies (self-blame, rumination, and catastrophizing), but not with sex of participants, extent of the skin lesion, and surgical removal of the nevus. Implications for clinical practice and future research are discussed.

## Introduction

Congenital melanocytic nevi (CMN) are pigmented birthmarks present at birth or appear during the first few weeks of life. CMN may occur as a single lesion or as multiple lesions located anywhere on the skin and they can vary in their morphological characteristics, such as color, surface, and hairiness (Jahnke et al., 2021). Incidences range from 1% to 6% for small to medium-sized CMN and up to 1 in 20,000 for large CMN with a projected adult size of ≥20 cm in diameter (Price and Schaffer, 2010). Most CMNs are clinically uncomplicated. However, some individuals experience mild to moderate physical symptoms, including pruritus and pain due to skin fragility (Neuhaus et al., 2020b). A few individuals suffer from severe and potentially life-threatening complications such as the development of melanoma and central nervous system pathology (Jahnke et al., 2021). Current management of CMN ranges from anticipatory counseling to surgical excision. In contrast to past practice, surgeries nowadays are mostly performed for aesthetic and psychosocial reasons, and melanoma prevention is only a very rare indication (Ott et al., 2019; Neuhaus et al., 2020a).

The potential impact of a visible skin condition on various aspects of health-related quality of life (HRQOL) and psychological adjustment has been well described (e.g., Dalgard et al., 2015; Rumsey, 2018). Individuals with CMN often experience unpleasant social reactions to their skin condition, including staring and intrusive questions, pity, avoidance, teasing, and bullying (Berg and Lindelof, 2002; Bellier-Waast et al., 2008; Masnari et al., 2012; Sampogna et al., 2021). This may cause significant emotional distress. Accordingly, previous studies have found an elevated risk of impairments of psychosocial health and psychological adjustment in children and adolescents with CMN (Koot et al., 2000; Masnari et al., 2019). However, data on HRQOL and psychological adjustment in individuals with CMN are still scarce. Previous studies are mostly based on parent reports of children with CMN (Koot et al., 2000; Masnari et al., 2019), with only limited findings based on self-reports of adolescents and young adults (Wramp et al., 2017; Neuhaus et al., 2020b).

Given the broad variance of adjustment in individuals with CMN (Wramp et al., 2017; Masnari et al., 2019; Neuhaus et al., 2020b), it is essential to investigate factors that contribute to resilience and distress. This would help to identify those at risk of maladaptive adjustment and to find relevant intervention targets.

Biopsychosocial models of adjustment to medical conditions suggest that individual adjustment depends on multiple factors, involving condition and treatment variables, family and societal factors, as well as individual factors, including cognitive processes and coping strategies (Thompson and Kent, 2001; Clarke et al., 2014; Masnari et al., 2015). Notably, findings in individuals with various visible differences (e.g., burns) consistently suggest that biomedical characteristics (e.g., extent of a skin condition or treatment variables) are often poorer predictors of individual adjustment compared to psychosocial factors, such as perceived stigmatization, social support, and coping strategies (Thompson and Kent, 2001; Noronha and Faust, 2007; Rumsey and Harcourt, 2007; Clarke et al., 2014). In parent reports of children and adolescents with CMN, impaired HRQOL and psychological adjustment have been found to be associated with lower socioeconomic status, higher age of child, malignant melanoma, neurological problems, skin-related discomfort, visibility of the skin lesion, and perceived stigmatization, but not with the size of the CMN and whether or not the CMN had been (partially) removed by surgery (Masnari et al., 2019; Neuhaus et al., 2020b). In a sample of 128 individuals with CMN (age range: 5–27, median age: 14 years) recruited through the Swedish medical birth register, 8% of respondents felt that the CMN caused bullying which they thought reduced their social activities (Berg and Lindelof, 2002). Some findings suggest that a scar might be socially more acceptable than a CMN (Koot et al., 2000), stigmatization and related psychosocial difficulties, however, may occur irrespective of whether the CMN was surgically removed or not (Sampogna et al., 2021). Finally, findings among individuals with various visible differences stress the importance of perceived social support (Thompson and Kent, 2001; Egan et al., 2011; Griffiths et al., 2012).

Psychosocial difficulties related to a visible difference depend on age and developmental phase (Rumsey and Harcourt, 2007). Living with a visible skin condition might be a particular burden in adolescence and emerging adulthood—a period characterized by a number of important developmental tasks, including identity exploration, developing friendships, achieving autonomy, identifying career goals, finding intimacy, and forming romantic relationships (Arnett, 2000). Stigma associated burden of living with a visible difference may interfere with these developmental tasks and be an additional concern during this already challenging life stage.

An interesting, new approach to investigate individual differences in psychosocial outcomes in this population might be to look at the role of emotion regulation strategies. A particularly powerful category of emotion regulation strategies involves the cognitive response to stressful life events (Garnefski and Kraaij, 2014). Various studies, including research among adolescents with a chronic medical condition (Kraaij and Garnefski, 2012), have consistently found that cognitive strategies such as self-blame, rumination, catastrophizing, and (inversely) positive reappraisal are associated with negative mental health outcomes, such as depression, anxiety, stress, and anger (e.g., Kraaij and Garnefski, 2012; Potthoff et al., 2016).

Taken together, current data on HRQOL and psychological adjustment in individuals with CMN are limited in several ways: the few findings published are mostly based on proxy reports, and more research is needed to identify possible predictors of individual adjustment. This is important for the development of evidence-based screenings and interventions. The aim of this study was, therefore, to assess self-reported HRQOL and psychological adjustment in adolescents and young adults with CMN and to explore associations with a wide range of variables which could affect individual outcomes. Reasoning from previous research, we hypothesized that impairments in psychosocial health and psychological adjustment were associated with lower SES, higher age of participants, visibility of the skin lesion, perceived stigmatization, low social support, and the following cognitive emotion regulation strategies: self-blame, rumination, catastrophizing, and (inversely) positive reappraisal. No direct association was expected for the extent of the skin lesion and whether the CMN was surgically removed or not.

## Materials and methods

### Procedures and participants

The data presented here are part of a comprehensive study assessing HRQOL, psychological adjustment, and treatment outcomes in children and adolescents with CMN (Masnari et al., 2019; Neuhaus et al., 2020a,b). This paper focuses on self-reports of adolescent and emerging adults with CMN. The Cantonal Ethics Committee of Zurich, Switzerland, waived approval for this study. Data were collected through a web-based survey. Informed consent was obtained electronically prior to commencing the survey. No personal identifying information was collected.

Participants were recruited worldwide with the help of CMN patient advocacy and support groups. Furthermore, participants were recruited among patients of the University Children’s Hospital Zurich by distributing leaflets to eligible families. Eligibility criteria were (1) self-reported diagnosis of a CMN, (2) age 14 to 25 years, and (3) sufficient command of English to complete the survey. Participants were included whether or not the CMN had been (partially) removed by surgery.

### Measures

#### Demographic information

Demographic information included participant’s age, sex, and country of residence. Socioeconomic status (SES) was assessed with the MacArthur Scale of Subjective Social Status, assessing subjective ratings of familial placement in society with a possible score ranging from 0 to 10, as proposed by Goodman et al. (2001). Three social classes were defined: *lower SES* (0–4), *middle SES* (5–7), and *upper SES* (8–10).

#### Location, extent, and visibility of the skin lesion

Respondents were presented a figure of a person, in which the body surface area was divided into 122 squares of the same size. They were asked to indicate how many squares were affected by the skin lesion. The number of affected squares was assessed separately for the following body parts: face, scalp, neck, collar, arms/shoulders, hands, chest, abdomen/flank, back/buttocks, and legs/feet. A score for the body surface area affected by the skin lesion was computed as the sum of all affected squares (BSA score; potential range 0–122). For correlation analyses, a dichotomous variable was used, indicating whether the skin lesion was located on highly visible body parts (face or hands) or not.

#### Surgical removal of CMN

Respondents were asked whether the CMN had been managed surgically. Possible answers were: 0 (*no surgery*), 1 (*some parts of the nevus skin have been removed, but most parts of the nevus skin have not been removed*), 2 (*most parts of the nevus skin have been removed, there is only little nevus skin left*), or 3 (*full removal of the CMN*). For correlation analyses, a dichotomous variable (“*CMN mostly not removed* (0–1)” vs. “*CMN fully/mostly removed* (2–3)”) was used.

#### Health-related quality of life

Health-related quality of life was measured with the Pediatric Quality of Life Inventory™ 4.0 (PedsQL™). This is a multi-dimensional, well-validated, generic instrument for assessing HRQOL. This study used two age-dependent versions (for ages 14–18 years and 18–25 years) of the self-report questionnaire (Varni and Limbers, 2009; Varni et al., 2011). The questionnaires cover four dimensions of functioning: physical, emotional, social, and cognitive/school functioning. Participants respond on a 5-point Likert scale, ranging from 0 (*never*) to 4 (*almost always*), with a recall period of 1 month. Scoring followed the standard PedsQL^™^ scoring protocol,^1^ and resulted in a score for each subscale and three summary scores: physical health, psychosocial health, and a total score. Higher scores indicate better HRQOL. In this study, internal consistency was good for all subscales, with Cronbach’s alphas between. 0.84 and 0.94. Means of study participants were compared with normative data derived from a population-based sample of 385 healthy individuals, ages 18–25 years, from a Dutch study (Limperg et al., 2014). This population-based sample was chosen because it was the best available match with the age range of our participants. The summary score “psychosocial health” was used as the outcome variable to examine possible associations with psychosocial HRQOL.

#### Psychological adjustment

Psychological adjustment was evaluated with the Strengths and Difficulties Questionnaire (SDQ): a brief, well-validated screening measure for emotional and behavioral problems (Goodman, 2001). The SDQ is widely used internationally in both low-and high-income settings (Achenbach et al., 2008). The SDQ questionnaire consists of 25 items grouped into four difficulties subscales (emotional, conduct, hyperactivity-inattention, and peer relationship problems) and a subscale on prosocial behavior. Participants respond on a 3-point Likert scale, including 0 (*not true*), 1 (*somewhat true*), and 2 (*certainly true*), with a recall period of 6 months. Following the standard SDQ scoring protocol,^2^ the four difficulty scales (score range: 0–10) were summed to generate a total score, and two broadband scales were computed (internalizing problems and externalizing problems). Higher scores indicate greater difficulties. The prosocial behavior scale was excluded from this study, since its items are not included in the total score nor in the two broadband scales (Goodman, 2001). The questionnaire used in this study was originally developed for the use in the U.S. among adolescents ages 11–17 years. We had chosen this questionnaire since the language of the survey was English and we expected many adolescent participants from the United States. We decided to use this questionnaire for the whole age range in our study (14–25 years) so that we could include all participants and compare outcomes within the whole group of participants. Internal consistency in this study was acceptable to good for the total SDQ score (*α* = 0.84) and internalizing difficulties (*α* = 0.80), but rather low for externalizing problems (*α* = 0.66). To provide clinically relevant classification, participants were categorized into three groups based on cut-off scores for 13–19-year-olds grounded on a large Norwegian population study (Van Roy et al., 2006). These cut-off scores are defined such that in the general population around 80% of adolescents score normal (low risk), 10% score borderline, and 10% score abnormal (high risk).

#### Perceived stigmatization and social support

The *Perceived Stigmatization Questionnaire* (*PSQ*) assesses a variety of stigmatizing behaviors commonly reported by individuals with a difference in appearance (Lawrence et al., 2006, 2010). The scale contains 21 items. Answer choices range from 1 (*never*) to 5 (*always*). A PSQ total score is obtained by averaging over all items. Higher scores indicate higher perceived stigmatization. In our study, internal consistency for the total score was very good (*α* = 95).

The *multidimensional scale of perceived social support by youth* assesses perceived social support in three subscales (family, friends, and significant other; Bruwer et al., 2008). The original scale has 12 items, with four items per subscale, rated on a 5-point Likert scale ranging from 1 (*strongly disagree*) to 5 (*strongly agree*). In this study, we chose six out of the 12 items, based on their factor loading in the study by Bruwer et al. (2008), including two items for each subscale. A total score was obtained by averaging over all items. Higher scores indicate higher perceived social support. In our study, internal consistency for the total score was very good (*α* = 91).

#### Cognitive emotion regulation strategies

Cognitive emotion regulation was assessed with the short form of the Cognitive Emotion Regulation Questionnaire (CERQ-short), which assesses cognitive emotion regulation strategies in response to unpleasant events or situations (Garnefski and Kraaij, 2006). The CERQ-short consists of 18 items, measuring nine conceptually distinct cognitive strategies, each of which is measured by two items. Items are measured on 5-point Likert scales ranging from 1 [(*almost*) *never*] to 5 [(*almost*) *always*]. To keep the overall survey as short as possible, we only included the following four strategies: self-blame, rumination, catastrophizing, and positive reappraisal. These four strategies were selected because previous studies have found that these strategies correlate significantly with psychopathology and especially with symptoms of depression and anxiety (Kraaij et al., 2003; Garnefski and Kraaij, 2007; Kraaij and Garnefski, 2012). Internal consistency was acceptable for most scales (self-blame *ρ* = 0.79, catastrophizing *ρ* = 0.88, and positive reappraisal *ρ* = 0.75) but questionable for the rumination scale (*ρ* = 0.62).

### Statistical analyses

SPSS software version 27 was used for data analysis (IBM Corporation, 2016). All analyses were performed with two-sided tests, and a *p* < 0.05 was considered significant. Scale internal consistency reliability was assessed by Cronbach’s alpha for scales containing more than two items and Spearman–Brown reliability estimates for two-items scales (Eisinga et al., 2013). One-sample *t-*tests were computed to compare means in our group of participants with reference data for the PedsQL^™^ scales. Mean differences were quantified by calculating effect sizes with Cohen’s *d*: 0.2 representing a small, 0.5 medium, and 0.8 large effect size (Cohen, 1988). Spearman’s correlations were computed to analyze associations between possible predictors and the psychosocial health scale of the PedsQL^™^ and the total score and the two broadband scales of the SDQ as outcome variables. Bonferroni correction was conducted to protect multiple analyses from type 1 error.

## Results

### Characteristics of study participants

Characteristics of study participants are described in Table 1. Many participants (44%) were from Europe (including France, Germany, Italy, Netherlands, Norway, Spain, Switzerland, and the United Kingdom), 28% were from the United States, 23% indicated another country (including Australia, Brazil, Canada, Israel, Mexico, Morocco, New Zealand, Philippines, and South Africa), and two participants preferred not to state their country of residence. Table 1 also presents information on characteristics of the CMN and mean scores of perceived stigmatization, social support, and the cognitive emotion regulation scales.

**Table 1:** Characteristics of participants (*N* = 43).

Variable	
**Age, years**	
Mean (*SD*), Range	17.56 (2.24), 14–24
**Sex**	
Female	36 (84%)
Male	7 (16%)
**Ethnicity**	
African American	1 (2%)
Asian or Pacific Islander	1 (2%)
European/European American/White	32 (74%)
Hispanic or Latino	4 (9%)
Middle Eastern	2 (5%)
Multi-Racial	3 (7%)
**Socioeconomic status, Mean (*SD*)**	6.63 (1.75)
Lower (0–4)	4 (9%)
Middle (5–7)	27 (63%)
Upper (8–10)	12 (28%)
**Location of the skin lesion**	
Face	15 (35%)
Hands	5 (12%)
Scalp	13 (30%)
Neck	10 (23%)
Collar	7 (16%)
Arms/shoulders	12 (28%)
Chest	9 (21%)
Abdomen	19 (44%)
Back	31 (72%)
Legs/feet	29 (67%)
Genitals	10 (23%)
**BSA score**	
Mean (*SD*), Range	16.44 (20.88), 1–106
**Surgical excision of CMN**	
No surgery	14 (33%)
Some parts of the nevus skin have been removed, but most parts have not been removed	15 (35%)
Most parts of the nevus skin have been removed, there is only little nevus skin left	9 (21%)
Full removal of CMN	5 (12%)
**Perceived social reactions**	
Perceived stigmatization, Mean (*SD*)	2.24 (0.77)
Perceived social support, Mean (*SD*)	5.21 (1.44)
**Cognitive emotion regulation strategies**	
Self-blame, Mean (*SD*)	1.91 (1.17)
Rumination, Mean (*SD*)	2.88 (1.12)
Catastrophizing, Mean (*SD*)	2.11 (1.14)
Positive reappraisal, Mean (*SD*)	3.44 (1.10)

CMN, congenital melanocytic nevus; BSA, body surface area affected by skin lesion.

### Health-related quality of life

Table 2 shows the mean scores of the PedsQL^™^ scales for study participants and the population-based reference group. Participants in our study reported lower psychosocial health than the reference group, with significant lower scores on emotional and social functioning. The largest effect size was found for emotional functioning (Cohen’s *d* = 0.75). Physical health was not impaired compared to the reference group.

**Table 2:** Means of study participants and reference data for self-reported health-related quality of life outcomes (PedsQL™ scales).

PedsQL™ scales	Study participants				Population-based norms			Comparison of groups	
	*n*	α^1^	*M*	*SD*	*n*	*M*	*SD*	*p*	*d*
Total score	43	0.94	75.46	17.49	385	83.82	12.74	<0.01	0.55
Physical health	43	0.86	84.38	16.54	385	87.78	15.16	0.18	0.21
Psychosocial health	43	0.93	70.70	20.16	385	81.71	13.66	<0.001	0.64
Emotional functioning	43	0.86	60.70	24.27	385	76.73	18.07	<0.001	0.75
Social functioning	43	0.90	76.05	23.92	385	86.64	14.90	<0.01	0.53
School functioning	43	0.84	75.35	20.86	385	81.75	15.45	0.05	0.35

1 Cronbach’s alpha.

### Psychological adjustment

Table 3 shows the mean scores of the SDQ scales and the clinical categorization based on population norms. For the SDQ total difficulties score, 24% of our study participants reported a score above clinical cut-offs, and 9% borderline scores. The prevalence of emotional problems was considerably higher in our study compared to population norms, with 31% of participants indicating a clinically relevant score and 19% a borderline score. The prevalence of peer problems was also higher, with 24% of participants indicating high-risk and 21% borderline scores.

**Table 3:** Means for self-reported strengths and difficulties (SDQ Scales) and clinical categorization based on cut-off points.

SDQ scales	Study participants				Categorization based on population-based cut-off scores, *n (%)*		
	*n*	α^1^	*M*	*SD*	Normal (low risk)	Borderline	Abnormal (high risk)
Total difficulties	42	0.84	12.79	6.79	29 (69%)	3 (7%)	10 (24%)
Internalizing scale	42	0.80	7.55	4.46			
Emotional problems	42	0.72	4.43	2.60	21 (50%)	8 (19%)	13 (31%)
Peer problems	42	0.69	3.12	2.42	23 (55%)	9 (21%)	10 (24%)
Externalizing scale	42	0.66	5.24	3.15			
Conduct problems	42	0.45	2.10	1.54	33 (79%)	5 (12%)	4 (9%)
Hyperactivity	42	0.54	3.14	2.07	39 (93%)	2 (5%)	1 (2%)

1 Cronbach’s alpha.

### Associations with psychosocial health and psychological difficulties

Table 4 shows Spearman’s correlations between possible predictor variables and outcome variables. Lower SES was significantly associated with lower psychosocial health and more internalizing problems as well as a higher total SDQ score. The age of participants was significantly associated with the SDQ total difficulties score. The extent of the skin lesion and whether the CMN had been surgically removed or not were not significantly associated with any outcome variable. Visibility of skin lesions was correlated with lower psychosocial health and more internalizing problems. Perceived stigmatization was associated with impairments in all outcome variables. Perceived social support, on the other hand, was associated with better psychosocial health and psychological adjustment. As regards cognitive emotion regulation strategies, self-blame, rumination, and catastrophizing were associated with negative psychosocial outcomes, whereas no significant association was found with positive reappraisal.

**Table 4:** Spearman’s correlations between predictor variables and outcome variables.

	Health-related quality of life		Psychological adjustment					
	Psychosocial health		Total difficulties score		Internalizing problems		Externalizing problems	
	*r*	*p*	*r*	*p*	*r*	*p*	*r*	*p*
**Sociodemographic variables**								
Age	−0.47	<0.01^*^	0.34	0.03	0.29	0.07	0.25	0.11
Male sex	−0.09	0.55	−0.15	0.36	−0.13	0.41	−0.15	0.33
SES	0.41	<0.01	−0.48	<0.01^*^	−0.51	<0.001^*^	−0.25	0.12
**Characteristics of CMN**								
Surgical removal of CMN	0.28	0.07	−0.22	0.16	−0.20	0.20	−0.25	0.11
BSA score	−0.14	0.39	0.00	0.99	0.06	0.69	−0.05	0.73
Visibility of skin lesion	−0.33	0.03	0.29	0.06	0.33	0.03	0.13	0.41
**Perceived social reactions**								
Perceived stigmatization	−0.60	<0.001^*^	0.61	<0.001^*^	0.62	<0.001^*^	0.34	0.03
Perceived social support	0.44	<0.01	−0.46	<0.01^*^	−0.56	<0.001^*^	−0.15	0.37
**Cognitive emotion regulation**								
Self-blame	−0.50	<0.001^*^	0.64	<0.001^*^	0.59	<0.001^*^	0.48	<0.01^*^
Rumination	−0.41	<0.01	0.47	<0.01^*^	0.46	<0.01^*^	0.35	0.02
Positive reappraisal	0.27	0.09	−0.21	0.20	−0.25	0.11	−0.09	0.58
Catastrophizing	−0.48	<0.01^*^	0.47	<0.01^*^	0.50	<0.001^*^	0.27	0.09

SES, socioeconomic status; CMN, congenital melanocytic nevus; BSA, body surface area affected by the skin lesion.

* *p* < the Bonferroni-corrected *p*-value of 0.004 (0.05/12).

## Discussion

The purpose of this study was to assess self-reported HRQOL and psychological adjustment in adolescents and young adults with CMN and to explore associations with a wide range of possible predictor variables.

In the HRQOL measures, participants reported lower psychosocial health compared to a population-based reference group, with significantly lower scores on emotional and social functioning. Physical health, however, was not impaired. These findings are in line with parent-reported HRQOL of adolescents with CMN (Masnari et al., 2019) and support the idea that HRQOL impairments in individuals with CMN are probably mostly caused by psychosocial difficulties rather than physical health problems. The broadband scale “psychosocial health” of the PedsQL^™^ combines the emotional, social, and school functioning items. These domains have some overlap with constructs that are assessed in the Strength and Difficulties Questionnaire (SDQ), which we used to assess psychological adjustment. According to the findings in the PedsQL^™^ scores, in the SDQ scores, the prevalence of emotional and peer problems was considerably higher in our group of study participants compared to population norms. Notably, 31% of participants scored a high-risk score for emotional problems, which is three times higher than in population norms. The prevalence of peer problems was 2.4 times higher than expected from population norms. These findings are in line with previous findings based on parents’ reports of children and adolescents with CMN (Koot et al., 2000; Masnari et al., 2019).

Increased psychosocial problems in individuals with CMN might be explained by several mechanisms: Stigmatizing experiences might lead to a negative self-image and feelings of shame and anxiety (Bellier-Waast et al., 2008). Affected individuals might react with maladaptive behavior (e.g., avoidance of potentially distressing situations or social withdrawal), which, again, may constrain their psychosocial development and well-being (Berg and Lindelof, 2002). Having a visible skin difference can therefore be considered a potential stressor, especially in vulnerable developmental phases. Findings in our study suggest that psychosocial impairments and psychological difficulties increase with higher age of participants. This could be because late adolescence/emerging adulthood might be a particular vulnerable phase in which the burden of living with a visible difference may interfere with important developmental tasks, such as establishing identity, identifying career goals, and enjoying romantic relationships. In a study among adolescents with psoriasis, participants reported concealing their condition from partners and avoiding intimacy (Fox et al., 2007). Similar avoidant behaviors were identified among adolescents and adults with a variety of skin conditions (Magin et al., 2010). As a result of low perceived attractiveness, self-consciousness, and fear of rejection, participants often felt that their appearance adversely impacted upon their romantic lives. This topic needs further examination in individuals with CMN.

Psychosocial health impairment, internalizing problems, and a higher SDQ total score were significantly associated with lower SES. This finding is in line with general child development literature which proposes a variety of mechanisms linking SES to child well-being (Bradley and Corwyn, 2002).

Whether the CMN was surgically removed or not and the extent of the skin lesion were not significantly associated with any outcome variable. This finding is in accordance with the general assumptions that biomedical characteristics of a condition are poorer predictors of adjustment compared to psychosocial factors (Noronha and Faust, 2007; Rumsey and Harcourt, 2007; Clarke et al., 2014). Notably, individuals with CMN have a high risk of experiencing stigmatization, irrespective of whether the CMN is surgically removed or not (Sampogna et al., 2021). Although some findings suggest that a scar might be socially more acceptable than a CMN (Koot et al., 2000), both forms of skin difference can result in perceived stigmatization and related psychosocial difficulties (Sampogna et al., 2021). Visibility of the skin lesion was significantly associated with lower psychosocial health and internalizing problems. It seems plausible that the effect of the visibility of the skin lesion on psychosocial outcomes might be influenced by perceived stigmatization, as was found in proxy reports of children with CMN (Masnari et al., 2019). Perceived stigmatization was significantly associated with impairments in all outcome variables, which is in line with previous findings (Masnari et al., 2013, 2019). Perceived social support, on the other hand, was associated with better psychosocial health and psychological adjustment. This is in line with the assumption that social support may act as a protective factor and lead to greater resilience (Thompson and Kent, 2001). Notably, social support might provide both a sense of being accepted and reassurance at times when people are subjected to humiliating reactions (Egan et al., 2011).

The individual emotional and behavioral reactions to experiences related to the skin condition are likely to depend on cognitive processes and emotion regulation strategies. Accordingly, we found significant correlations between psychosocial health and psychological adjustment with cognitive emotion regulation strategies. Notably, self-blame, rumination, and catastrophizing were all correlated with poorer outcomes. These results corroborate the findings of previous work in the field of cognitive emotional regulation and the assumption that individuals who tend to use these strategies may be more vulnerable to emotional problems than others (Kraaij and Garnefski, 2012; Garnefski and Kraaij, 2014; Potthoff et al., 2016).

We found no significant association between participants’ sex and any outcome variable. However, the unequal sex distribution in our group of participants (84% females) may have made it difficult to identify the effects of sex on outcome variables. Moreover, the lack of a direct effect of sex on the outcome variables does not exclude the possibility of an indirect effect through different use of emotion regulation strategies.

Taken together, our findings suggest that individual psychosocial health and psychological adjustment are significantly associated with age of participants, socioeconomic status, visibility of the skin lesion, perceived stigmatization, social support, and the following cognitive emotion regulation strategies: self-blame, rumination, and catastrophizing. Further research with a larger sample allowing multivariate analyses is needed in order to analyze the unique role of each predictor variable and possible mediation/moderation effects.

### Strengths and limitations

This is the first study analyzing associations between psychosocial and psychological outcomes with possible predictor variables in adolescents and young adults with CMN. Its strengths include a multi-national group of participants, an understudied population, the use of well-validated, multidimensional measures of HRQOL and psychological adjustment with population-based reference data, and analysis of a wide range of possible predictors. The widespread recruitment of participants through CMN support groups around the world has the advantage of reaching many individuals, irrespective of whether they seek medical treatment. However, such recruitment might lead to bias. The exclusion of non-English-speaking families and the use of a web-based survey may have caused an underrepresentation of families with lower SES. Also in our study female participants (84%) are overrepresented, this must be kept in mind when interpreting the results.

This study has further limitations: The cross-sectional design makes inferences about causal relations difficult, causational processes need to be examined longitudinally. Notably, the relationship between emotion regulation strategies and psychological difficulties could be bidirectional: not only could maladaptive strategies lead to psychological difficulties, but psychopathology might also cause the use of maladaptive strategies. Another limitation is the small sample size, which imposed some restrictions on statistical power. Notably, due to the rather small sample size, we were not able to perform regression analyses. Further research with more participants is needed to perform multivariate analyses, and to test the interaction effects of predictor variables. Moreover, our results for externalizing problems need to be interpreted with caution due to the poor internal consistency of the subscales in our study. The scale “rumination” also showed a rather low internal consistency. Finally, comparison with community norms should be interpreted with caution. Notably, the self-report version of the Strength and Difficulties Questionnaire used in this study was originally developed for adolescents ages 11–17 years, and clinical cut-off points are defined based on a population study among 13–19-year-old Norwegians, whereas our participants were aged 14–15 years. Moreover, one must keep in mind the international composition of our sample. It is unknown whether and how cultural differences may compromise comparability. However, it must be noted that whereas previous studies found significant cross-cultural differences in the use of cognitive strategies, the direction of the relationship between specific strategies and symptoms of psychopathology has been found to be consistent across countries (Potthoff et al., 2016). This supports the idea of a transcultural approach to identifying helpful and maladaptive coping strategies.

### Implications

The findings of this study have important implications for clinical practice, the development of evidence-based psychological interventions, and future research. It is important to note that participants in our study reported significant impairment of psychosocial health and increased emotional and peer problems. Therefore, comprehensive management of individuals with CMN should include not only medical but also psychological screening, and if needed, specific support. Due to the large interindividual differences in adjustment, psychosocial support should be provided within a stepped-care framework (Clarke et al., 2014).

Our study revealed strong associations between psychosocial health/psychological adjustment with SES, visibility of the skin lesion, perceived stigmatization, perceived social support, and the cognitive emotion regulation strategies. These findings may help to identify individuals at risk of maladaptive adjustment and to provide relevant targets for intervention. These might include the assistance in coping with perceived stigmatization, in seeking social support, and in reducing the use of maladaptive cognitive emotion regulation strategies while acquiring new, more adaptive strategies.

Finally, further research is needed with a larger sample and multivariate analyses in order to analyze the unique role of each predictor variable and possible mediation/moderation effects.

## Data availability statement

The raw data supporting the conclusions of this article will be made available by the authors, without undue reservation.

## Ethics statement

Ethical review and approval was not required for the study on human participants in accordance with the local legislation and institutional requirements. Written informed consent from the participants’ legal guardian/next of kin was not required to participate in this study in accordance with the national legislation and the institutional requirements.

## Author contributions

All authors were involved in the conceptualization of this study. OM carried out the survey, performed the computations, and took the lead in writing the manuscript. ML supervised the work and verified the analytical methods. All authors contributed to the article and approved the submitted version.

## Funding

This work was supported by a grant from Nevus Outreach Inc. (www.nevus.org) - an association for CMN and related disorders. This funder had no role in study design, data analysis, decision to publish, or preparation of the manuscript. Open access publishing fees were covered by University Library Zurich.

## Conflict of interest

This study received funding from Nevus Outreach Inc. (http://www.nevus.org) - an association for CMN and related disorders. The funder helped with the promotion of this study as did other CMN patient organizations in order to reach as many individuals with CMN as possible. All authors declare no other competing interests.

## Publisher’s note

All claims expressed in this article are solely those of the authors and do not necessarily represent those of their affiliated organizations, or those of the publisher, the editors and the reviewers. Any product that may be evaluated in this article, or claim that may be made by its manufacturer, is not guaranteed or endorsed by the publisher.

## Footnotes

^1^ www.pedsql.org

^2^ www.sdqinfo.org
